# Unlocking the potential of immunotherapy in platinum-resistant ovarian cancer: rationale, challenges, and novel strategies

**DOI:** 10.20517/cdr.2024.67

**Published:** 2024-10-15

**Authors:** Joanna Kefas, Michael Flynn

**Affiliations:** Medical Oncology, University College London Hospitals NHS Foundation Trust, London NW1 2PG, UK.

**Keywords:** Ovarian cancer, immunotherapy, immune checkpoint inhibition, immunotherapy resistance

## Abstract

Ovarian cancer is a significant global health challenge, with cytoreductive surgery and platinum-based chemotherapy serving as established primary treatments. Unfortunately, most patients relapse and ultimately become platinum-resistant, at which point there are limited effective treatment options. Given the success of immunotherapy in inducing durable treatment responses in several other cancers, its potential in platinum-resistant ovarian cancer (PROC) is currently being investigated. However, in unselected advanced ovarian cancer populations, researchers have reported low response rates to immune checkpoint inhibition, and thus far, no validated biomarkers are predictive of response. Understanding the intricate interplay between platinum resistance, immune recognition, and the tumour microenvironment (TME) is crucial. In this review, we examine the research challenges encountered thus far, the biological rationale for immunotherapy, the underlying mechanisms of immune resistance, and new strategies to overcome resistance.

## INTRODUCTION

Ovarian cancer is the 7th most common cancer and the 8th most common cause of cancer-related deaths in women globally^[1]^. In 2020, an estimated 314,000 new cases and 207,000 deaths were reported, with global incidence expected to increase by 42% by 2040. Ovarian cancer encompasses a heterogeneous group of diseases, including ovarian, fallopian tube, and primary peritoneal cancers. Histologically, 90% of ovarian cancers are of epithelial origin, with high-grade serous ovarian carcinoma (HGSOC) being the most common subtype. Less common subtypes include clear cell, endometrioid, low-grade serous ovarian carcinoma (LGSOC), mucinous, and other rarer variants. On a molecular and genetic level, ovarian cancer displays even greater heterogeneity. The main first-line treatments are cytoreductive surgery and platinum-based chemotherapy^[2,3]^. More recent treatment advances have led to the approval of the vascular endothelial growth factor inhibitor (VEGFi) bevacizumab, as well as poly(adenosine diphosphate-ribose) polymerase inhibitors (PARPis)^[4-6]^.

The typical progression of epithelial ovarian cancer (EOC) involves an initial favourable response to platinum-based chemotherapy, followed by prognostically significant platinum-free intervals. However, most patients eventually develop platinum resistance, characterised by disease recurrence either during or immediately after their final round of platinum-based chemotherapy^[2]^. At this stage, single-agent non-platinum-based chemotherapy regimens can be considered, though they offer a low likelihood of durable response, with median progression-free survival (PFS) of 3 to 4 months and no demonstrated improvement in median overall survival (OS) of 12-18 months^[7-10]^. The effectiveness of platinum-based chemotherapy, due to its on-target DNA crosslinking, relies on DNA damage response pathways. For example, the reintroduction of platinum-based chemotherapy in triple-negative breast cancer follows the expected response to DNA-damaging interventions for BRCA-mutated and other homologous recombination repair (HRR) deficient breast cancers^[11]^. The upregulation or restoration of repair mechanisms, such as HRR and nucleotide excision repair, can lead to enhanced repair of platinum-induced DNA damage and, therefore, tolerance and resistance to platinum treatment^[12]^. Microsatellite instability has also been shown to confer resistance to platinum-based chemotherapy in preclinical models^[13,14]^. Pre-target dysregulation in drug uptake or efflux is another resistance mechanism. Although the exact link between transporters and resistance remains unclear, platinum-resistant cancers often show reduced intracellular drug accumulation. It was initially believed that platinum-based drugs enter cells passively through carrier proteins such as copper transporter 1 (CTR1)^[15]^. More recently, researchers have linked increased resistance, particularly in BRCA-1 mutated ovarian cancer cells, to the loss of specific volume-regulated anion channel (VRAC) subunits^[16]^. The impact of overexpressed efflux pumps, such as multidrug resistance-associated protein 2 (MRP2), may play an important role in some tumours^[17]^. Off-target altered tumour microenvironment (TME) by non-tumour cells, such as cancer-associated fibroblasts (CAF) and innate and adaptive immune cells, can have a protective effect on platinum-induced apoptosis, permitting drug resistance and cell survival^[15,18]^.

The biology of chemoresistance involves a complex interplay of tumour and host factors, making the development of effective treatments to overcome platinum-resistant ovarian cancer (PROC) a significant challenge. The role of immunotherapy in this setting is being explored, but studies thus far are largely negative, with low response rates compared to the current standard of care. In this review, we explore the rationale and existing shortfalls of immunotherapy in PROC. We also discuss the evolving immunotherapy landscape, the elements underlying immunotherapy resistance, and novel solutions.

## RATIONALE FOR IMMUNOTHERAPY IN OVARIAN CANCER

Single-agent immunotherapy in advanced ovarian cancer has shown response rates of up to 15%, which are much lower than those reported for tumours with drug approval, such as melanoma, lung cancer, head and neck squamous cell carcinoma, urothelial carcinoma, renal cell cancer, and cervical cancer^[19-21]^. However, some efficacy in EOC is suggested, as the observed disease control rates are 37%-52% and its durability in responders is 8-13 months.

The biological rationale for immunotherapy-based strategies stems from markers of immunogenicity in tumour-related genomic instability and extrinsic innate immune cells, adaptive immune cells and the TME, all of which can shift in the context of platinum resistance.

### Genomic instability, BRCA and homologous recombination

Ovarian carcinoma is a genomically complex cancer characterised by structural instability and DNA repair dysregulation, resulting in distinct TME profiles^[22,23]^. Mutational analysis has identified clusters with distinct signatures, and correlated prognostic differences and treatment responses based on BRCA-1/2 mutations, foldback inversions, intrachromosomal structural variations, and CDK12/tandem duplications^[24]^.

Fifty percent of HGSOCs exhibit homologous recombination deficiency (HRD) due to germline or somatic mutations in DNA repair mechanisms, most commonly in the *BRCA-1/BRCA-2* genes^[25]^. Defective HRR repair pathways are unable to repair double-strand breaks. The introduction of DNA damage-inducing or PARP inhibitor agents to HRD cells subsequently causes synthetic lethality via the accumulation of single-strand and double-strand DNA breaks. The effectiveness of PARP inhibition in HRD patients has been demonstrated in both frontline studies, such as SOLO1^[26]^, PAOLO1^[5]^, and PRIMA^[27]^, and platinum-sensitive relapsed settings, such as ARIEL2^[28]^, ARIEL3^[29]^, and NOVA^[30]^. A PFS benefit in HR-proficient patients was also observed in the PRIMA and ATHENA studies^[27,31]^. Detection of HRD in the Myriad myChoice next-generation-sequencing (NGS)-based assay is based on analysis of structural rearrangements in tumour DNA across the entire genome for three biomarkers for HRD: loss of heterozygosity, telomere imbalance, and large-scale transitions^[32-34]^. The combined biomarker scores determine the genomic instability score (GIS), with a cut-off of 42 and above indicating HRD positivity, as per the PRIMA and PAOLO-1 trials^[5,27]^. The Myriad myChoice assay, however, only reveals the *BRCA-1/2* mutation status and not the other *HR* gene mutations analysed, and this “scar” assay does not provide an indication of what is happening contemporaneously in the tumour.

HRD tumours have been shown to have higher neoantigen presentation and programmed death ligand-1 (PD-L1) expression, with distinct spatial TMEs that may make them more immunogenic^[35,36]^. Genetically modified mouse models demonstrate that HRD tumours are more responsive to immunotherapy, particularly when combined with PARPi or platinum-based chemotherapy^[37,38]^. The case for combining immunotherapy with PARPi is not solely due to its direct cytotoxic activity but also its antitumour immunity properties via the stimulator of interferon genes (*STING*) pathway, which could complement and augment immune checkpoint blockade^[39-43]^. However, thus far, ovarian cancer patients with BRCA-1/2 mutations, HRD positivity, or high tumour mutational burden (TMB) have not shown improved responses to immune checkpoint inhibitors (ICPIs)^[44]^.

### Innate immune cells

The innate immune cell types include natural killer (NK) cells, eosinophils, basophils, mast cells, neutrophils, monocytes, macrophages, and dendritic cells. They have direct killing properties and trigger adaptive immune responses^[45]^. Uniquely, NK cells eliminate cancer cells by secreting cytotoxic granules governed by activating and inhibitory signals through mechanisms independent of tumour antigens. Although platinum-mediated and NK cell-mediated cytotoxicity is induced by distinct mechanisms, platinum-resistant cells have also been found to be resistant to NK cell killing and increases in the number of NK cells influence sensitivity to cisplatin therapy^[46]^. As first responders in the immune system, neutrophils acquire a tumour-promoting phenotype, advancing tumour proliferation, invasion, and angiogenesis. A high neutrophil-to-lymphocyte ratio is an independent prognostic indicator in patients with ovarian cancer and is also associated with platinum resistance^[47,48]^. Tumour-associated macrophages (TAMs) are important components of the innate system with variable capabilities and roles in immune surveillance and eradication. They coordinate immune responses through pathogen phagocytosis, antigen presentation, and cytokine secretion. TAMs can exhibit an anti-inflammatory M2 phenotype that promotes tumour growth, angiogenesis, epithelial-mesenchymal transition, extracellular matrix remodelling, and the secretion of anti-inflammatory cytokines such as CD206/CD68 and IL-10^[49]^. The resulting overall effect promotes cell survival and chemoresistance. In cell lines, macrophage phenotype transformation is closely related to the development of platinum resistance^[50]^.

Dendritic cells serve as a crucial link between the innate and adaptive immune systems, playing a key role in antigen presentation and T cell activation. In cancer, tumour-infiltrating dendritic cells demonstrate tumour-specific variations in dendritic cell phenotype, composition, and functionality, ranging from immunosuppressive to antitumour activity. Chemotherapy agents can induce an immunogenic type of cell death mediated by dendritic cells, but how this crossover effect relates to chemoresistance is not well understood^[51]^.

### Adaptive immune cells

In the adaptive immune system, tumour-infiltrating lymphocytes (TILs) constitute a central host response mechanism with oligoclonal expansion, tumour antigen recognition, and cytotoxic capabilities^[52]^. They have become a widely explored TME component of interest for prognostic and predictive value. Histopathological evaluation of TILs can be performed through haematoxylin and eosin (H&E)-based immunohistochemistry (IHC) and immunofluorescence (IF) and scored on all mononuclear cells including lymphocytes and plasma cells^[53]^. T lymphocyte subsets, including CD8+ cytotoxic and CD4+ helper lymphocytes, play distinct roles based on surface molecules or cytokine production^[54]^. While CD8+ T cells generally enhance antitumour immunity, regulatory cells such as Tregs and Bregs often suppress these responses and promote tumour growth. TIL subsets vary not only between ovarian cancer histological subtypes. Variable TIL population ratios can be seen within the same patient in different anatomical locations and between primary *vs.* recurrent tumours^[55]^. In 2003, Zhang *et al.* conducted an IHC analysis of 186 advanced ovarian carcinoma specimens and detected the presence of TILs in 54.8% of tumours and favourable outcomes after surgical debulking and adjuvant chemotherapy^[56]^. Compared with TIL-absent patients, the presence of CD3+ TILs was associated with an almost 4-fold increase in PFS and OS. In those with a complete response to treatment, the presence of TILs was associated with a 10-fold increase in PFS. Multivariate analysis also revealed an independent association with delayed recurrence or delayed death. In a subsequent meta-analysis of 10 studies and 1,815 patients, the absence of intraepithelial TILs (CD3+ or CD8+) was again a significantly poor prognostic marker^[57]^. A further systematic review of 19 studies and 6,004 patients confirmed that CD3+, CD4+, CD8+, and CD103+ TILs are reliable biomarkers for OS and PFS in HGSOC^[58]^. Similarly, more recent translational research in cutaneous melanoma and early breast cancers has demonstrated that high TILs are predictive of response (dependent on subtype biology) and offer a favourable survival benefit independent of staging^[59-62]^. Meta-analyses have also shown a predictive and prognostic value of TILs in oesophageal, colorectal, and pancreatic cancers^[63-65]^.

The presence and subtype of TILs can significantly impact patient prognosis and response to treatment, especially in ovarian cancer, in which a relatively high ratio of CD8+ T cells to Tregs is associated with improved survival. Compared with CD3 positivity, CD8 positivity has a stronger and more reproducible association with better survival^[66]^. The co-localisation of CD20+ B cells with T cells further enhances the positive prognostic impact of TILs^[67,68]^. In comparison, regulatory T cells, usually identified by FOXP3 and CD25 co-expression, have immunosuppressive qualities that can be exploited by tumour cells to create immune-privileged sites^[69]^. However, the prognostic significance of CD25+ FOXP3+ Tregs is controversial, as both positive and negative impacts on survival have been reported^[70,71]^. There are also inconsistencies in predictive and prognostic value when evaluating EOC subtypes and whether TILs are usually located in the intraepithelial *vs.* in the stromal compartment^[72-74]^. The clinical impact of the localisation, spatial distribution, density, and clonal subpopulation of TILs is still only partially understood.

Activated TILs increase their expression of programmed death 1 (PD-1), which represents a hallmark of T cell exhaustion^[75]^. PD-1 bound to PD-L1/2 ligands expressed on tumour cells impairs the cytotoxic capabilities of TILs. Tumours exploit these inhibitory immune checkpoint mechanisms to avoid immune surveillance^[76]^. Relevant biomarkers correlated with clinical response in approved settings are PD-L1 IHC-scored expression, TMB, and mismatch repair (MMR)^[77]^. TMB is a biomarker for neoantigen load and a well-recognised predictor of response to immunotherapy across various solid tumours^[78,79]^. Mismatch repair deficiency (dMMR) is the only tumour-agnostic predictive biomarker for response to pembrolizumab^[80,81]^. dMMR is often associated with high mutational burden and CD8+ infiltration, although both can occur in MMR-proficient tumours. Unlike immune-responsive cancers and even other gynaecological cancers, EOCs are typical of a TMB-low phenotype and are microsatellite stable^[82]^. In PROC studies, PD-L1 expression is estimated to be between 33% and 80%^[83-85]^.

Drawing a comparison of PD-L1 expression and its predictive value between trials is challenging, as staining methods with respect to different antibodies, biomarker platforms, cut-offs, and main epitope locations differ. In the context of platinum-resistant disease, another challenge is the use of archival *vs.* de novo metastatic site tissue for PD-L1 staining. The tumour proportion score (TPS) reflects the percentage of tumour cells with membranous PD-L1 expression, whereas the combined positive score (CPS) includes PD-L1 stained tumour cells, lymphocytes, and macrophages as a proportion of the total number of tumour cells. In numerous studies, the presence of TILs has been correlated with PD-L1 expression on tumour cells^[86-88]^. However, no association has been found between PD-1+ TILs and survival^[58,89]^. Although a higher CPS has been associated with improved responses, overall responses, if present, are observed regardless of PD-L1 expression.

In the neoadjuvant setting, platinum chemotherapy has demonstrated significant changes in the TME, enhancing the adaptive immune response with oligoclonal T cell expansion, increasing CD4+ cytolytic activity, but also increasing the levels of the inhibitors PD-1, PD-L1, and cytotoxic T-lymphocyte associated protein-4 (CTLA-4)^[90]^. In the advanced setting, tumours often become “cold” owing to changes in the TME characterised by less infiltration of CD8+ T lymphocytes, increased infiltration of Tregs, low mutational burden, low neoantigen expression, and low PD-L1 status^[44,91]^. Tumour analysis at the point of treatment resistance is needed. While the change in B cells during platinum resistance is less well understood, it is expected that tumour-infiltrating B cells enrich memory cell-mediated antigen presentation and, therefore, have a positive effect on the immune response and outcomes.

### Nonimmune TME

In addition to innate and adaptive immune cells, the TME comprises stromal cells, fibroblasts, and endothelial cells. There are limited data on the changing TME in recurrent PROC. The coordinated but complex interaction between innate and adaptive immune cells and nonimmune cells within the TME can shift in the context of platinum resistance in a way that contributes to tumour progression and mechanisms of drug resistance, making overcoming platinum resistance challenging.

## IMMUNOTHERAPY CLINICAL TRIALS SO FAR IN OVARIAN CANCER

To date, immunotherapy with PD-1 or PD-L1 blockade has shown limited efficacy in ovarian cancer in both the first-line and recurrent settings. Over the last 10 years, key phase I/II and phase III studies in untreated advanced disease and platinum-resistant disease have explored the therapeutic options of immunotherapy as monotherapy, dual checkpoint inhibition, or in combination with chemotherapy and targeted therapies, all yielding mixed results that underscore the complexity of identifying effective treatment combinations [Table 1].

**Table 1:** Key clinical trials so far of immunotherapy in advanced ovarian cancer

**Study**	**Phase**	**Population**	**Participants**	**Immunotherapy agent**	**Comparator arm**	**Intervention arm(s)**	**Findings**	**Outcome**	**Potential predictive biomarkers**
JAVELIN Ovarian 100 NCT02718417	3	Untreated stage III-IV epithelial ovarian, fallopian tube, or primary peritoneal cancer	998	Avelumab	Chemotherapy alone (Carboplatin-paclitaxel)	Chemotherapy in combination with avelumab followed by avelumab in maintenance Chemotherapy followed by avelumab maintenance	mPFS 18.1 mo (HR 1.14) mPFS 16.8 mo (HR 1.43)	Negative trial	None
JAVELIN Ovarian 200 NCT02580058	3	Platinum-resistant or -refractory ovarian cancer	566	Avelumab	PLD alone	Avelumab alone Avelumab in combination with PLD	mOS 11.8 mo (HR 1.14) mPFS 1.9 mo (HR 1.68) mPFS 3.7 mo (HR 0.78) mOS 15.7 mo (HR 0.89)	Negative trial	PD-L1-positive (SP263) CD8
NINJA	3	PROC	316	Nivolumab	Chemotherapy alone (GEM or PLD)	Nivolumab alone	mOS 10.1 mo (HR 1.0) mPFS 2.0 mo (HR 1.5)	Negative trial	Clear cell carcinoma
NRG GY003 NCT02498600	2	Persistent or recurrent epithelial ovarian, fallopian tube or primary peritoneal cancer	100	Nivolumab and ipilimumab	Nivolumab	Induction nivolumab and ipilimumab followed by maintenance nivolumab	ORR 31.4% (OR 3.28) mPFS 3.9 mo (HR 0.53)	Positive trial	Clear cell carcinoma Platinum resistance
IMAGYN050 NCT03038100	3	Untreated stage III-IV ovarian, fallopian tube, or primary peritoneal cancer	1,301	Atezolizumab	Placebo with paclitaxel, carboplatin and bevacizumab	Atezolizumab with paclitaxel, carboplatin and bevacizumab	mPFS 19.5 mo (HR 0.92)	Negative trial	PD-L1 > 5% (SP142) Clear cell carcinoma
TOPACIO/KEYNOTE-162 NCT02657889	1/2	Platinum-resistant advanced or metastatic epithelial ovarian, fallopian tube, or primary peritoneal cancer	62	Pembrolizumab	None	Pembrolizumab in combination with niraparib	ORR 18% DCR 65% mPFS 3.4 mo	Negative trial	tBRCAmut Sig3 IS
OPAL NCT03574779	2	Platinum-resistant or refractory ovarian cancer in cohort A	41	Dostarlimab (TSR-042) and bevacizumab	None	Dostarlimab (TSR-042), bevacizumab, and niraparib	ORR 17.9% DCR 76.9% mPFS 7.6 mo	Positive trial	None
MOONSTONE NCT03955471	2	BRCAwt PROC with previous bevacizumab use	41	Dostarlimab (TSR-042)	None	Dostarlimab (TSR-042) in combination with niraparib	ORR 7.3%	Negative trial	None
PEACOCC NCT03425565	2	Advanced clear cell gynaecological cancer	48	Pembrolizumab	None	Pembrolizumab	PFS rate at 12 weeks 43.8% ORR 25.0% 1-year DOR 47.7%	Positive trial	None

mPFS: Median progression-free survival; mo: months; HR: hazard ratio; PLD: pegylated liposomal doxorubicin; mOS: median overall survival; PD-L1: programmed death ligand-1; PROC: platinum-resistant ovarian cancer; GEM: gemcitabine; PLD: pegylated liposomal doxorubicin; ORR: objective response rate; OR: odds ratio; DCR: disease control rate; tBRCAmut: tumour BRCA mutation; Sig3: mutational signature 3; IS: immune score; BRCAwt: BRCA wild-type; DOR: duration of response.

### Immunotherapy in advanced disease regardless of platinum sensitivity

The JAVELIN Ovarian 100 trial investigated a potential upfront benefit of anti-PD-L1 avelumab, either in combination with carboplatin/paclitaxel or as maintenance therapy, compared to chemotherapy alone in 998 randomised patients^[92]^. At the planned interim analysis, the trial was discontinued due to futility, as no PFS benefit was observed. The stratified hazard ratio (HR) surprisingly favoured the control group, as the PFS was 1.43 (95%CI 1.05-1.95, *P* = 0.99) with avelumab maintenance and 1.14 (95%CI 0.83-1.56, *P* = 0.79) with the avelumab combination, compared to the control. The safety profile was as expected and similar between the groups. The patient population was an unselected treatment-naïve group, and at the time of study enrolment, the BRCA status was incomplete, and HRD was not assessed. With early termination and treatment discontinuation, OS data were immature. Subsequent subgroup analyses revealed no predictive biomarkers based on PD-L1, CD8, and germline BRCA1/2 status^[93]^. The BRCA-mutant subgroup did better than the BRCA-wildtype subgroup, but the addition of avelumab had no added advantage.

For dual immune checkpoint pathway inhibition, the NRG GY003 phase II trial randomised 100 patients with platinum-sensitive and platinum-resistant recurrent ovarian cancer to single-agent nivolumab (3 mg/kg every 2 weeks) or induction nivolumab plus anti-CTLA-4 ipilimumab (nivolumab 3 mg/kg and ipilimumab 1 mg/kg every 3 weeks, for 4 doses) followed by maintenance nivolumab (every 2 weeks, for up to 42 doses)^[94]^. In the primary analysis, there was a significantly greater overall response and disease stability with dual checkpoint therapy than with nivolumab alone (odds ratio 3.28, 85%CI 1.54 to infinity, *P* = 0.034). Dual treatment had a favourable median OS of 28.1 *vs.* 21.8 months (HR 0.79, 95%CI 0.44-1.42, *P* = 0.43), and the median PFS was 3.9 *vs.* 2 months, favouring dual treatment (HR 0.53, 95%CI 0.34-0.82, *P* = 0.004). In the exploratory analysis, clear cell carcinoma and PROC subsets had a better response to dual treatment, and there was no association between PD-L1 status and PFS. Specifically in the PROC cohort, although similar to known safety profiles, the frequency of grade 3 treatment-related adverse events (TRAEs) was 49% with dual treatment and 33% with nivolumab alone. Overall, this was a small study with limited power, and any responses seen were not durable, nor did patients in the combination arm experience significantly improved survival.

Trials have also assessed combining immune checkpoint inhibition with VEGFi or PARPi, given their established efficacy in ovarian cancer. VEGF inhibition has been used effectively in combination with platinum-taxane chemotherapy for first-line ovarian cancer and was approved in PROC based on the AURELIA trial^[95-97]^. VEGFi use in combination with ICPI has shown clear efficacy in other solid tumours such as metastatic non-small cell lung cancer and unresectable hepatocellular carcinoma^[98-100]^. In the first-line setting, the IMagyn050 randomised phase III trial compared carboplatin-paclitaxel with bevacizumab plus placebo *vs.* bevacizumab plus atezolizumab in 1,301 patients^[101]^. This was a negative trial with no PFS or OS benefit with atezolizumab in the intention-to-treat (ITT) or the PD-L1 > 1% expressing population. However, in preplanned exploratory analyses, a significant PFS was seen in the population with PD-L1 expression greater than > 5% (HR 0.64, 95%CI 0.43-0.96); this subgroup represented 20% of the VENTANA SP142 IHC PD-L1-positive cohort. Although the sample size was small, the subgroup with the greatest improvement in PFS was the clear cell histological types. BRCA and HRD status were not available during randomisation, but recently published biomarker analyses found neither BRCA1/2 mutation nor HRD was predictive of response to atezolizumab^[102]^. Irrespective of HRD status, 97% of all tumours had a low TMB, and there was no correlation between BRCA mutations and PD-L1 status.

### Immunotherapy in platinum-resistant disease

The JAVELIN Ovarian 200 randomised phase III trial also failed to meet PFS and OS endpoints when comparing avelumab alone *vs.* combination with pegylated liposomal doxorubicin (PLD) and PLD alone in 566 patients with PROC^[85]^. The median PFS for combination avelumab plus PLD *vs.* PLD alone was 3.7 *vs.* 3.5 months (HR 0.78, 95%CI 0.59-1.25, *P* = 0.030). The median OS for combination therapy *vs.* PLD alone was 15.7 *vs.* 13.1 months (HR 0.89, CI 0.74-1.24, *P* = 0.21). The confirmed objective response rates (ORRs) were 13% in the combination group and 4% in both the PLD and avelumab monotherapy groups. In the planned subgroup analyses, PD-L1 and CD8 expression levels were predictive of treatment response to combination avelumab and PLD. A total of 57% of participants had PD-L1-positive tumours, and with combination treatment, the unstratified HR was 0.65 (0.46-0.92) for PFS and 0.72 (0.49-1.05) for OS. A total of 46% of participants had CD8-positive tumours, and with combination treatment, the unstratified HR was 0.64 (0.44-0.95) for PFS and 0.66 (0.44-0.95) for OS. In the subgroup that was both PD-L1- and CD8-positive, combination treatment was favoured with an unstratified HR 0.53 (0.34-0.83) for PFS and 0.53 (0.32-0.89) for OS. Again, the BRCA status of the included patients was incomplete and not evaluated. The median duration of follow-up was less than 2 years, so we are unable to comment on potential long-term efficacy. Reflective of the poor prognosis in PROC, within 2 months of randomisation, 50% of patients either died, progressed, or withdrew from the study.

Following phase II efficacy data of nivolumab in PROC, the randomised phase III NINJA trial evaluated anti-PD-1 nivolumab (240 mg every 2 weeks) *vs.* physician-choice gemcitabine (1,000 mg/m^2^ days 1, 8 and 15 of a 28-day cycle) or PLD (50 mg/m^2^ every 4 weeks) in 316 patients^[103]^. However, nivolumab was not superior to gemcitabine or PLD in terms of the primary endpoint OS (10.1 *vs.* 12.1 months, HR 1.0, 95%CI 0.8-1.3, *P* = 0.808). The median PFS was worse with nivolumab treatment (2.0 *vs.* 3.8 months, HR 1.5, 95%CI 1.2-1.9, *P* = 0.002). Although there was no significant difference in overall response rates between groups, of those who did respond, the median duration of response was 18.7 months with nivolumab *vs.* 7.4 months with gemcitabine or PLD. In the preplanned subgroup analysis, there was no difference based on PD-L1 score, but there was a trend towards longer OS with nivolumab in clear cell subtypes (HR 0.78, 95%CI 0.46-1.32). Nivolumab was well tolerated, with a lower incidence of any-grade TRAEs and treatment discontinuations. Patient-reported quality-of-life outcome measures were similar between the groups.

ICIs combined with PARPis have shown widely variable response rates. The TOPACIO/KEYNOTE-162 single-arm phase I/II trial combined pembrolizumab (200 mg IV every 3 weeks) with niraparib (200 mg once daily) in platinum-resistant, platinum-refractory, and non-platinum eligible patients^[104]^. A total of 62 patients were enrolled, irrespective of BRCA mutation status. The ORR was 18% overall and 21% in the platinum-resistant subgroup, and the predefined statistical criteria for this study were not met. Exploratory analyses of BRCAm or HRD positivity were not identified as predictive biomarkers of response. Subsequent exploratory immunogenomic profiling of trial participant samples using a signature multivariate analysis (SigMA) tool was able to identify two previously unknown predictive biomarkers, mutational signature 3 (Sig3) and positive immune score (IS), as surrogates of HRD and interferon-primed exhausted CD8 T cells, respectively^[105]^. If either was present, PFS was significantly increased (HR 0.32, 95%CI 0.15-0.70, *P* = 0.002). Sig3 and IS were absent in all non-responders.

Similarly, the phase II OPAL trial of 41 patients with PROC combined niraparib with dostarlimab and bevacizumab as triplet therapy^[106]^. The ORR was 18%, and although there were no complete response events, 76.9% of patients achieved disease control. Also evaluating dostarlimab and niraparib, the phase II single-arm MOONSTONE trial in BRCA-wildtype PROC was terminated at interim analysis due to futility, with an ORR of 7.3%^[107]^.

The negative studies and limited efficacy observed so far may be due to a lack of identifying robust predictive biomarkers. This leads to challenges with patient selection, as does inadequate stratification of factors such as BRCA status, HRD, or specific immune profiles. Subgroup analyses show positive trends in treatment response for clear cell carcinoma subtypes and particular “immunoscore” expression profiles^[101,103,104]^. Relevant to the clear cell subgroup, the PEACOCC phase II single-arm trial investigated pembrolizumab monotherapy in advanced clear cell gynaecological cancers^[108]^. A total of 43.8% of patients achieved disease control at 12 weeks. The median PFS was 12.2 weeks, and the median OS was 16 months. Translational analysis of archived and mandatory fresh biopsies is awaited.

Other reasons for disappointing outcomes are that some trials may have suffered from suboptimal designs, including inadequate endpoints, insufficient power, or inappropriate control arms. Well-designed studies that incorporate biomarker-driven strategies are needed. The study design and methodology also need to review the choice of trial endpoints and plan longer follow-up periods, as delayed separation of survival curves can occur. Direct trial comparisons of the predictive value of PD-L1 are limited because of the different scoring systems and testing methodologies used, as there can be varying cut-offs, inclusion of expressive cell types, and temporal and spatial heterogeneity. To date, there remain no validated predictive biomarkers of immunotherapy response. Future trials should, therefore, look beyond ICPIs to other mechanisms of immune evasion that can be actively or passively leveraged.

## MECHANISMS OF RESISTANCE TO IMMUNOTHERAPY

**Figure 1:**
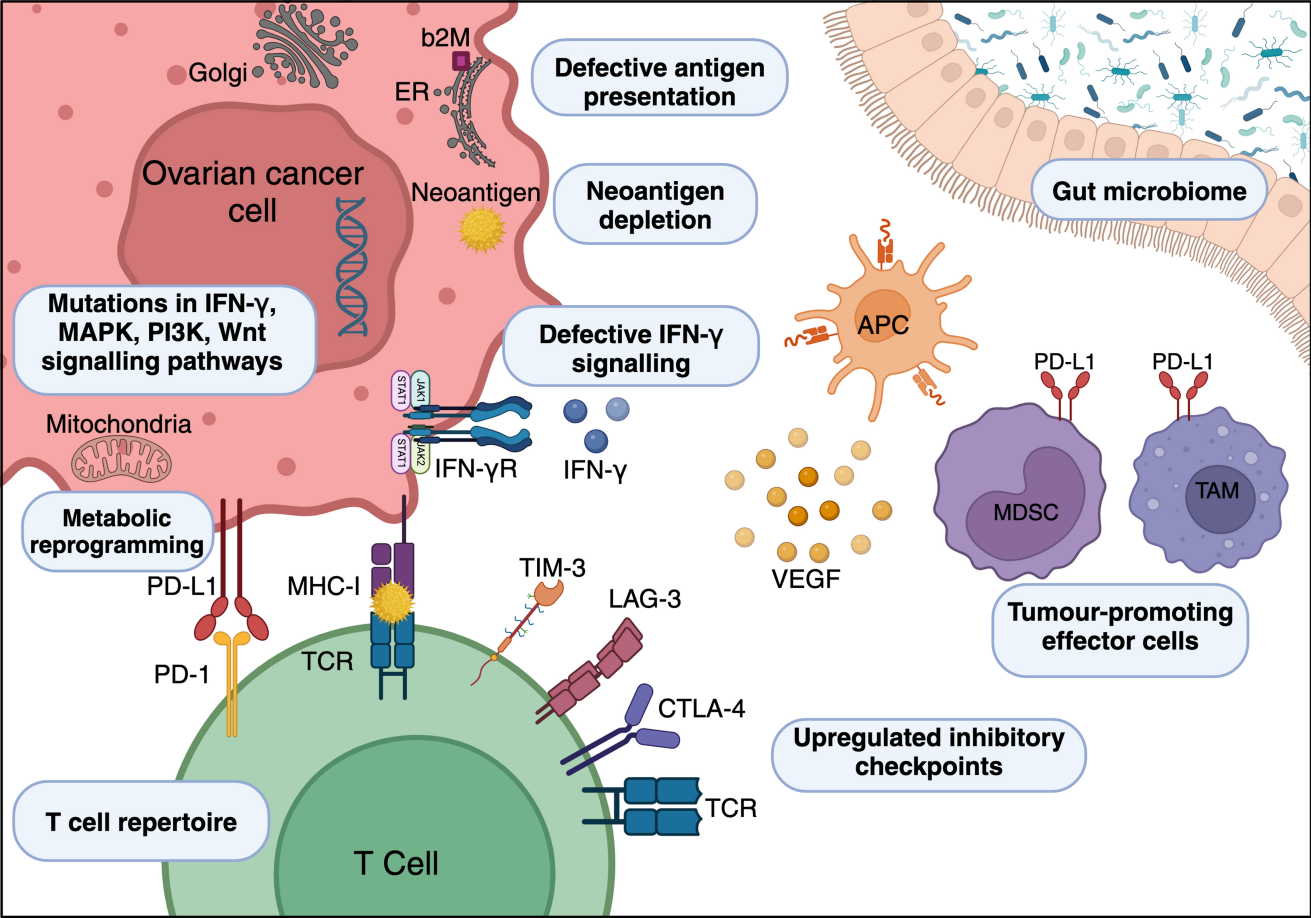
Mechanisms of immunotherapy resistance. Schematic representation of immunotherapy resistance mechanisms in ovarian cancer. Mechanisms intrinsic to the ovarian cancer cell are defective antigen presentation, neoantigen depletion, altered signalling pathways, and metabolic reprogramming. Extrinsic to the cancer cell are T cell repertoire, upregulated inhibitory checkpoints, tumour-promoting effector cells, and the gut microbiome. b2M: Beta-microglobulin; ER: endoplasmic reticulum; Golgi: Golgi apparatus; MHC-I: major histocompatibility complex I; TCR: T cell receptor; TIM-3: T cell immunoglobulin and mucin domain 3; LAG-3: lymphocyte activation gene 3; CTLA-4: cytotoxic T-lymphocyte-associated protein 4; APC: antigen-presenting cell; PD-L1: programmed cell death ligand 1; PD-1: programmed death 1; TAM: tumour-associated macrophages; MDSC: myeloid-derived suppressor cell; VEGF: vascular endothelial growth factor; IFN-γ: interferon-gamma; IFN-γR: interferon-gamma receptor; MAPK: mitogen-activated protein kinase; PI3K: phosphoinositide 3-kinases; Wnt: wingless-related integration site; JAK: Janus kinase; STAT: signal transducer and activator of transcription. Created with BioRender.com.

Despite the radical shift in cancer treatment paradigms with the adoption of immunotherapy, most patients still do not benefit. Resistance to immunotherapy can be primary, referring to a lack of initial response, or acquired, where there is clinical and/or radiological progression after an initial response to therapy. Overcoming resistance requires an understanding of the underlying resistance mechanisms, whether intrinsic or extrinsic to tumour cells [Figure 1].

### Intrinsic mechanisms of resistance

#### Tumour intrinsic loss of major histocompatibility complex

Immune recognition and T cell activation are dependent on antigen presentation by major histocompatibility complexes (MHCs) on antigen-presenting cells (APCs). Genetic variants such as single nucleotide polymorphisms and epigenetic modifications of *HLA* gene expression, or other aspects of the antigen presentation machinery alter function and impair antigen presentation pathways. For example, a resulting loss of MHC-I heterozygosity creates less diverse peptide-binding specificities, narrows immune recognition, and has been associated with reduced responses to ICPI^[109]^. In a cohort study of over 1,500 patients with advanced cancer, maximal heterozygosity at HLA-I loci (“A”, “B”, and “C”) significantly improved OS after ICPI therapy compared with patients who were homozygous for at least one locus^[110]^. In baseline and progressive melanoma tumour sampling, acquired resistance was associated with loss-of-function mutations in beta-2 microglobulin (b2M), a stabilising component of HLA class I complexes^[111]^. This led to the downregulation of *MHC class I* genes and a failure to present tumour neoantigens. Antigen presentation and immune evasion can also be affected by epigenetic modifications within cancer cells, including DNA methylation, histone modification, and noncoding RNA^[112]^. There is preclinical evidence that reversing these changes can restore immune checkpoint responses^[113]^. A second component of antigen recognition is T cell receptors (TCRs), whose diversity and clonality are also associated with immunotherapy efficacy^[114,115]^. However, TCR and antigen recognition mechanisms are complex and change over time and in response to various treatment modalities. The significance of sequencing of the TCR repertoire as a potential biomarker at baseline or during treatment remains unknown.

#### Insufficient antigenicity

Tumour-specific neoantigens arising from somatic mutations serve as highly specific targets for immune effector cells. The expression of neoantigens and self-antigens is one of the primary determinants of tumour immunogenicity and response. Antigenicity correlates with mutational burden, which is often considered the reason why tumours with higher TMB, such as melanoma, lung cancer and bladder cancer, tend to have stronger immune checkpoint responses. Primary resistance occurs with neoantigen depletion due to clonal selection, epigenetic repression, copy number loss, and transcript repression^[116]^. Acquired resistance emerges through tumour evolution and treatment-related changes to the neoantigen repertoire^[117,118]^. Nonsynonymous mutations appear more relevant than total exonic mutation burden in determining immunotherapy response, likely due to their resulting protein conformation changes^[119]^.

#### Intrinsic signalling and metabolic pathways

In response to neoantigen recognition, effector T cells release interferon-gamma (IFN-γ), triggering a signalling cascade that mediates *MHC-I* gene and PD-L1 expression via Janus kinase (JAK) 1 and 2 pathways and the phosphorylation of signal transducer and activators of transcription (*STATs*) and *IFN-γ* stimulated genes^[120]^. Defective IFN-γ mutations are a distinct immune escape mechanism associated with poor responses^[121]^. A potential solution, therefore, is to activate other signalling pathways, bypassing the need for functioning IFN-γ signalling pathways and altering the TME through stimulation of pro-inflammatory cytokines and NK cell recruitment^[122]^. Similarly, alterations in Wnt/B-catenin signalling are also linked to resistance. Wnt is one of the major developmental signalling pathways involved in embryonic development, cell proliferation and differentiation. Dysregulated Wnt/B-catenin signalling is associated with suppression of CD8+ T cell infiltration and CD4+ T cell differentiation and resistance to immune checkpoint inhibition^[123]^. In preclinical models, loss of phosphatase and tensin homolog (PTEN) constitutively activates the phosphatidylinositol 3-kinase (PI3K) signalling pathway, which is associated with a reduction in TILs, IFN-γ expression and T cell activation, along with an increase in immunosuppressive cytokines and VEGF, which overall is inhibitory to T cell infiltration and function^[124]^. Besides being pro-angiogenic, VEGF has immunosuppressive properties primarily by inhibiting dendritic cell maturation, reducing T cell tumour infiltration, and promoting inhibitory effector cells myeloid-derived suppressor cell (MDSC) and T regs^[98,125]^. VEGFi may reverse its immunosuppressive effects and offer a potential combination treatment strategy.

In addition to intracellular signalling, metabolic alterations to increase aerobic glycolysis, increase glutamine metabolism, and activate the pentose phosphate pathway and fatty acid synthesis can alter the TME by recruitment shifting towards an immunosuppressive type^[126,127]^. Metabolic by-products exert immunosuppressive effects by inhibiting the function of T cells and NK cells. Potentially, metabolic inhibitors such as glutamine antagonists and mitochondrial inhibitors in combination with immunotherapy could improve treatment outcomes^[128,129]^.

### Extrinsic mechanisms of resistance

Tumour-extrinsic resistance can occur due to upregulated coinhibitory checkpoint receptors such as CTLA-4, TIM-3, and LAG-3, expression of immunoregulatory cytokines, and promotion of antitumour activity of immune effector cells such as MDSCs and M2-phenotype TAMs which have been linked to clinically relevant immunosuppression and poor disease outcome in HGSOC^[130-132]^. MDSCs dampen NK cell activity, regulate T cell differentiation, and generate M2-phenotype macrophages through mediators such as arginase 1, inducible nitric oxide synthase, and reactive oxygen species^[133]^. Drugs reducing MDSCs in animal models were able to restore susceptibility to immune checkpoint inhibition^[134,135]^.

Adding further to the complexity of the TME, the gut microbiome and its relationship to immunotherapy resistance is of expanding interest, given its emerging immunomodulatory role. Gut microbiome profiles, such as those with a higher abundance of *Faecalibacterium prausnitzii* and *Akkermansia muciniphila*, have been associated with ICPI response^[136,137]^. The use of antibiotics within 2 months before or the month after the first immunotherapy dosing had an independent negative effect on PFS and OS, likely owing to changes in the composition of the gut microbiome^[137]^. There is a potential therapeutic opportunity to modulate the gut microbiome with probiotics, prebiotics, antibiotics, dietary interventions, and faecal microbiota transplantation to improve treatment outcomes. It may not be necessary to modify the microbiome itself, as antibody-directed strategies, such as those used by Park *et al.*, can overcome gut microbiome-dependent resistance mechanisms^[138]^. However, at this stage, challenges remain in understanding the complexity and in identifying reliable biomarkers and targets.

Resistance to immunotherapy involves multiple overlapping mechanisms, interactions and cross-talk between tumour cells, immune cells, and stromal cells within the TME. Determining the contribution of each component *in vivo* remains problematic. Most clinical trials to date have not targeted patient-specific resistance mechanisms.

## NEXT STEPS: STRATEGIES TO OVERCOME RESISTANCE

Lessons can be drawn from the advances and challenges of immunotherapy in different cancer types that have shown sustained clinical responses. In 2011, ipilimumab, an anti-CTLA-4 antibody, was the first immune checkpoint blockade therapy to receive FDA approval^[139]^. Since then, multiple breakthroughs have led to the approval of other inhibitors targeting CTLA-4, PD-1, PD-L1, and LAG-3 pathways for clinical use. While these therapies have achieved significant successes, including durable responses even in cases of dose-limiting toxicities or early treatment discontinuation, immune checkpoint inhibition is not universally effective. Many patients either do not respond to treatment or experience only a short-term benefit.

Approaches to enhance efficacy have centred on inducing a pro-inflammatory state, inducing immunogenic cell death, and recruiting antigen-presenting cells. This is achieved through combination strategies with concurrent chemotherapy and radiotherapy, or combination targeted blockade.

Priming the immune system against tumour antigens has been explored using oncolytic viruses. These viruses induce direct tumour lysis, leading to the release of tumour-associated antigens, which in turn foster both local and systemic immune-stimulatory environments. Talimogene laherparepvec (T-VEC), a first-in-class genetically modified type 1 herpes simplex virus, has demonstrated significant and durable responses. Intralesional administration of T-VEC has produced high overall response rates in patients with unresectable or advanced melanoma, with these effects sustained in long-term follow-up studies^[140]^.

Among the phase 3 immunotherapy studies in PROC that yielded negative results, many had robust study designs. However, only two (JAVELIN 200 and IMAGYN 050) included PD-L1 as an exploratory biomarker^[85,92,101,103]^. One possible reason for the lack of success is that the complex biology of the disease, which leads to inherent immunotherapy resistance in most patients, may not have been fully considered during the design of these studies. Consequently, they did not incorporate stratification based on emerging biomarkers, such as elevated TILs or downregulated signalling pathways, which may confer resistance in PROC.

Another approach to overcoming resistance is through prospective stratification and patient selection in biomarker-guided adaptive studies. Scoring PD-1 expression and microsatellite stability helps determine the suitability of immune checkpoints for non-small-cell lung cancer, and head and neck squamous cell carcinoma, and microsatellite instability-high colorectal cancer^[141-143]^. As personalised medicine evolves, there is an opportunity to identify novel biomarkers and evaluate their efficacy within biomarker-adaptive trial designs. This may enable patients to access effective treatments earlier in their therapeutic journey^[144]^.

### Dual antibodies and other immunomodulators

Bispecific antibodies represent a type of immunotherapy that can simultaneously target two different antigens or epitopes. Ubatamamb is a human bispecific antibody that binds to overexpressed mucin 16 glycoprotein (MUC16) on ovarian cancer cell surfaces and CD3-activated T cells^[145]^. By binding both cancer and host immune T cells, it bridges and facilitates T cell recognition and elimination of MUC16-expressing cancer cells. Immune-deficient mouse models have demonstrated dose-dependent antitumour activity. A first-in-human phase I/II dose-escalation study of Ubatamab (REGN4018) as monotherapy or in combination with anti-PD-1 cemiplimab is currently recruiting [NCT03564340]. Preliminary phase I efficacy results presented at ESMO 2022 showed an ORR of 14% and a disease control rate of 57% in patients who received at least one dose over 20 mg^[146]^. The estimated median duration of response was 12.2 months. The safety profile and tolerability are acceptable, and the most common TRAEs are cytokine release syndrome and pain with the first four doses. GEN1047 (Duobody-CD3×B7H4) is another bispecific antibody currently undergoing a first-in-human phase I-II study, which includes a cohort of PROC patients expressing B7H4 [NCT05180474].

Studies investigating myeloid immune checkpoints such as CD47 within the TME may overcome the limited efficacy seen with T cell immune checkpoint inhibition^[147,148]^. Similarly, myeloid checkpoints can be targeted with monoclonal or bispecific antibodies. CD47 is highly expressed in EOC and forms a signalling complex with signal-regulatory protein alpha (SIRa)^[149,150]^. The SIRPa-Fc-CD40L ARC fusion protein (SL-172154) is a bifunctional fusion protein that blocks CD47/SIRPa on tumour cells and CD40/CD40L on immune antigen-presenting cells. Following a safety and tolerability phase 1 monotherapy study^[151]^, a phase 1b study combining SL-172154 with either PLD or Mirvetuximab soravtansine (MIRV) is enrolling [NCT05483933]. The PLD arm was chosen based on its established use as a standard of care in PROC. MIRV is a DM4 payload-delivering antibody-drug conjugate that targets folate receptor-a (FRa). MIRV did not meet its primary PFS endpoint in either the ITT or FRa-positive population in the FORWARD1 trial^[152]^, but demonstrated superiority over standard-of-care chemotherapy in secondary endpoints of ORR, Ca-125 response, and patient-reported outcomes. More recently, it received FDA approval following the results of the patient-selected SORAYA trial, in which there was an ORR of 36% for FRα-high patients with PROC^[153]^.

Preferentially expressed antigen in melanoma (PRAME) is an appealing target for immunotherapy-based treatments, given its high expression in various tumours and limited expression in normal tissue^[154]^. Immune-mobilising monoclonal T cell receptors against cancer (ImmTAC) are a new class of T cell-based bispecific fusion proteins with high-affinity T cell receptors that target presented peptide-HLA complexes^[155]^. Its clinical efficacy as tebentafusp (gp100-directed ImmTAC) has been approved and validated in uveal melanoma^[156]^. IMC-F106C is the first PRAME×CD3 ImmTAC and targets HLA-A2-presented peptides from PRAME-expressing tumour cells. The IMC-F106C phase I-II study is currently recruiting [NCT04262466] and preliminary results presented at ESMO 2022 demonstrate durable disease responses and stabilisation in the heavily pretreated PROC patient cohort, as well as manageable toxicities and evidence of early biomarker circulating tumour DNA (ctDNA) response^[157]^.

A re-emerging treatment of interest due to its immunostimulatory effects is interleukin-2 (IL-2) therapy. High-dose IL-2 was the first immunotherapy ever approved^[158]^. Recent advancements in selectivity are addressing previous concerns with significant TRAEs^[159]^. ARTISTRY studies of Nemvaleukin alfa, a novel engineered selective IL-2 fusion protein, revealed that it stimulates NK cells and CD8+ T cells and potentially switches “cold” tumour phenotypes to “hot”. In the Phase 1 ARTISTRY-1 trial PROC cohort, the ORR was 28.6%, and the disease control rate (DCR) was 71.4%, with two complete responses and two partial responses among the 14 patients^[160]^. Subsequently, ARTISTRY-7, a phase 3 trial testing Nemvaleukin alfa in combination with pembrolizumab against standard-of-care chemotherapy, has opened, and the data read-outs are pending [NCT05092360].

Concurrent or sequential combination therapies such as doublet or triplet treatment strategies may augment immune checkpoint inhibition. Zsiros *et al.* have completed recruitment in a phase II single-arm study of triplet pembrolizumab, bevacizumab, and oral cyclophosphamide in both platinum-sensitive and platinum-resistant recurrent ovarian cancer^[161]^. The potential effects of oral cyclophosphamide on the TME include increasing antigen presentation and downregulating Tregs. The ORR was 47.5% overall and 43.3% in the PROC cohort, with an overall median PFS of 10 months. Notably, all the complete responders had platinum-resistant disease. Although most patients included in the study had PROC, this is the highest reported response rate in this cohort and represents a promising combination strategy worth further exploration.

### PARP inhibition

The search for an effective combination of PARPis and immunotherapy continues, as a PARPi-primed TME is biologically plausible to be more immune-favourable, owing to its higher neoantigen load and the induction of type 1 interferons and pro-inflammatory cytokines^[162]^. Numerous upfront trials with dual or triplet combinations are in progress and translational work will hopefully identify biomarkers predictive of response and define future trial selection^[163-168]^.

### Cancer vaccines

Cancer vaccines are used prophylactically or therapeutically and can be further subcategorised based on mechanism of action and type of antigen target. Therapeutic cancer vaccines activate the host adaptive immune system and achieve tumour regression by targeting either tumour-specific antigens or tumour-associated antigens, with the benefit of eradicating disease with minimal off-target toxicities^[169,170]^. The development of cancer vaccines surged following the COVID-19 pandemic; however, it remains largely in the early-phase trial stage. A phase I first-in-human study of the OVM-200 therapeutic survivin-targeted vaccine included a PROC cohort [NCT05104515]. As a member of the inhibitor of apoptosis (IAP) family, survivin negatively regulates apoptosis and programmed cell death. In cancer, survivin is commonly upregulated, expressed in over 70% of ovarian cancers. Its expression is of particular interest as it correlates with chemotherapy resistance^[171,172]^.

Another survivin pathway targeting trial with Maveropepimut-S (MVP-S), a potent inducer of survivin-specific T cell responses, reported early efficacy in phase I/II data, indicating a clinical benefit regardless of platinum sensitivity. The AVALON trial of MVP-S and low-dose cyclophosphamide in patients with PROC (NCT0524324) has completed recruitment.

Other ovarian cancer-associated antigens as potential therapeutic vaccine targets include human epidermal growth factor receptor 2 (HER-2/neu), MUC1, Ca-125, FR-a, p53-synthetic long peptide, Wilms’ tumour 1 (WT1) peptide, and New-York-oesophageal squamous cell carcinoma-1 (NY-ESO-1)^[173]^. The challenge is not only to identify the correct antigen but also to select suitable formulations, adjuvants, and delivery methods. Personalised cancer vaccines targeting specific tumour antigens based on individual tumour profiles are being explored.

### Adoptive cellular therapy

Adoptive T cell therapy utilises *in vitro* genetically modified immune cells such as TILs and has its foundations in haematological malignancies. The rationale is to transfer specifically primed T cells with potent high-affinity cytotoxic activity. This technique holds exciting prospects for treating refractory and resistant diseases with high durability. The ongoing SURPASS phase I trial is evaluating the outcomes of autologous ADP-A2M4CD8, a next-generation specific peptide enhanced affinity receptor (SPEAR) engineered to target melanoma-associated antigen A4 (MAGE-A4) in an HLA-A2 antigen complex [NCT04044859]. The latest safety and efficacy data from the phase I SURPASS study presented at ESMO 2022 report an overall response rate of 31% and a disease control rate of 75.9% with ADP-A2M4CD8 monotherapy in all tumour groups^[174]^. Based on this, the trial has moved forward with a dedicated ovarian cohort, known as the SURPASS-3 trial, with ADP-A2M4CD8 as monotherapy or in combination with nivolumab in PROC [NCT05601752]. However, trials for adoptive T cell therapy are costly, time-consuming, and technically challenging, as they involve screening, pretreatment conditioning, treatment delivery, and the management of adverse events. Given these challenges, adoptive cell therapy is not widely available.

### Chimeric antigen receptor T cell therapy

Chimeric antigen receptors can recognise tumour antigens independent of major histocompatibility complex expression. It is an innovative treatment approach and an area of active research in solid tumours. Genetically engineered T cells specific to tumour-associated antigens have been successful in haematological malignancies but more challenging in solid tumours such as ovarian cancer in both preclinical and clinical studies^[175]^. Ovarian cancers often lack tumour-specific antigens, but there are several tumour-associated antigenic targets, including erb-b2 receptor tyrosine kinase 2 (ERBB2), programmed cell death-ligand, anti-Müllerian hormone receptor type 2 (AMHR2), and MUC16^[176-179]^. Several challenges remain, including off-target toxicity, heterogeneity of ovarian tumours and their TME, T cell persistence and exhaustion, and limited access to these investigational products due to the complexity and cost of studies. Intraperitoneal administration may be a safe route to minimise on-target off-tumour toxicity and increase persistence and migration to tumour sites.

### Radiotherapy

Ionising radiation can not only damage DNA, but also increase neoantigen load and alter the TME. An abscopal effect in which local irradiation induces a systemic antitumour effect at distant sites beyond the irradiated field can also occur^[180]^. The exact mechanisms remain unknown but are likely immune-mediated. This, therefore, raises the possibility of combining treatments to augment the abscopal effect. The phase III PACIFIC trial of anti-PD-L1 durvalumab after concurrent chemoradiotherapy led to a beneficial mPFS of 16.9 *vs.* 5.6 months (HR 0.55, 95%CI 0.45-0.68) and mOS of 47.5 *vs.* 29.1 months (HR 0.72, 95%CI 0.59-0.89)^[181]^.

In advanced EOC, radiotherapy is primarily used palliatively due to the excellent efficacy and tolerability of platinum-based chemotherapy^[182,183]^. However, its more indirect immunostimulatory properties could lead to its repurposing as an immunomodulatory treatment with synergistic or radio-sensitising doses to reprogramme the TME to a more immune-favourable phenotype. For example, the recruiting SOPRANO phase II randomised study is exploring disease activity with stereotactic body radiation therapy (SBRT) alone *vs.* SBRT followed by niraparib in patients who develop oligometastatic or oligoprogressive disease while receiving a PARP inhibitor [NCT05990192].

## CONCLUSION

Despite the biological plausibility for the role of immunotherapy in PROC, immunotherapy studies to date - including those involving combination chemotherapy or targeted therapies - have been disappointing, with no validated predictive biomarkers available. Our understanding of mechanisms underlying platinum resistance and immune recognition in ovarian cancer is limited. Further studies deconstructing the relationship between ovarian cancer genetic dysregulation, immune recognition, and the TME are needed. Prioritised areas of investigation should focus on validating predictive and real-time response biomarkers, such as specific gene expression profiles and immune cell signatures. To establish their predictive value, prospectively validated biomarkers must be assessed through preplanned biomarker analyses in randomised controlled trials. The complexity of cancer and the evolution of its microenvironment with subsequent treatment lines renders analysing archival tissue alone insufficient. However, the development and integration of circulating tumour DNA sequencing will enable future trials to conduct real-time analyses as patients undergo treatment.

As our understanding of various cell types in the TME advances, we should trial multitarget treatments such as metabolic reprogramming, microbiome modulation, and alternative inhibitor checkpoints. Personalised approaches that address specific resistance mechanisms through vaccine therapy may provide additional insights. Biomarker-guided adaptive trial designs would focus resources on informed patient stratification and adaptive randomisation, allowing for trial modifications based on interim futility analyses. This will enhance the likelihood of identifying effective treatments and accelerate the development of personalised therapies.

Exploratory subgroup analyses suggest that certain subpopulations may benefit from ICPIs with durable responses demonstrated. The correlation between PROC and immunotherapy resistance may indicate that PROC has an inherent “cold” immune phenotype. TIL infiltration, PD-1/PD-L1 expression, HRD, and high TMB do not consistently correlate with response to the immunotherapy. Improved patient selection and the use of therapies that modulate the TME may improve treatment outcomes.

## DECLARATIONS

### Copyright

© The Author(s) 2024.
